# Physical activity and mental health in sports university students during the COVID-19 school confinement in Shanghai

**DOI:** 10.3389/fpubh.2022.977072

**Published:** 2022-10-11

**Authors:** Yufei Wang, Youqiang Li

**Affiliations:** School of Physical Education, Shanghai University of Sport, Shanghai, China

**Keywords:** COVID-19, confinement, physical activities, mental health, sports university students

## Abstract

**Background:**

In 2022, Shanghai was seriously affected by the coronavirus disease 2019 (COVID-19) pandemic. The government implemented citywide static management for 2 months, as well as all universities in Shanghai, which changed the normal learning and living style of sports students and led to a decline in physical activity level. As the physical activity has a strong correlation with mental health, this study aimed to investigate the current state of physical activity (PA) and mental health of the students in Shanghai University of Sport. It will try to reveal the correlation between PA and depressive symptoms, anxiety symptoms, fear of COVID-19 and smartphone addiction.

**Methods:**

A cross-sectional survey was conducted on a random sample of 400 students who came from six different majors in May 2022 at the Shanghai University of Sport. Respondents completed the International Physical Activity Questionnaire Short Form (IPAQ-SF), the Chinese version of the 9-item Patient Health Questionnaire (PHQ-9), the Chinese version of the Generalized Anxiety Disorder Scale (GAD-7), the Chinese version of the COVID-19 Fear Scale (FCV- 19S), and the Smartphone Addiction Scale (SAS-SV). Demographics, PA, depressive symptoms, anxiety symptoms, fear of COVID-19, and smartphone addiction were compared. A binary logistic regression model was used for the further analysis.

**Results:**

A total of 376 college students were included in the final analysis. Binary logistics analysis showed that moderate physical activity (MPA) was negatively correlated with depression (OR = 0.95, 95%CI = 0.93–0.98), anxiety (OR = 0.97, 95%CI = 0.95–0.99), fear of COVID 19(OR = 0.99, 95%CI = 0.98–0.99)and smartphone addiction (OR = 0.94, 95%CI = 0.9–0.98) (all *P* < 0.05). Sedentary behavior was positively correlated with smartphone addiction (OR = 1.01, *P* < 0.01, 95%CI = 1.001–1.004).

**Conclusion:**

There was an association between the presence of MPA and depressive symptoms, anxiety symptoms, fear of COVID-19, smartphone addiction, and sedentary behavior associated with smartphone addiction levels. Clarifying the causal relationship between PA and mental health will require further research.

## Introduction

In late February 2022, a round of SARS-CoV-2 infection broke out in Shanghai. According to the Shanghai Health Commission, as of May 4, 2022, there have been more than 593,336 cumulative cases of SARS-CoV-2 infection, of which 538,450 were asymptomatic, with 503 cumulative deaths (1). COVID-19 spread rapidly on a large scale, leading to strict and comprehensive pandemic control strategies throughout the city for 2 months. The lockdown resulting in closing public places, primary and secondary schools. Universities implemented school closures and residents implemented a policy of quarantine at homes under strict control and isolation-related measures that the epidemic was effectively controlled. However, this dramatic lifestyle change might affect the wellbeing of different age groups (2). According to previous studies, measures including restriction of movement and isolation can lead to varying degrees of increased mental health problems. For instance, the prevalence of major depressive disorder and anxiety disorders during the COVID-19, respectively, reached 27.6 and 25.6%, with much higher prevalence than before the COVID-19 (3). A Canadian study of anxiety and depression symptoms in adults during the COVID-19 also found that the proportion of respondents with high to very high levels of anxiety increased from 5 to 20%, and the number of respondents reporting high levels of depression increased from 4 to 10% (4). Not only that, confinement and lockdown also tended to cause problems such as insufficient physical activities, increased sedentary behavior, excessive use of electronics, and increased fear of COVID-19 infection (5–8). Moreover, the increase of these undesirable behaviors and mental health problems may further aggravate the symptoms of depression and anxiety in the epidemic. It may seriously affect people's mental health (9–11).

As a group in society vulnerable to mental health disorders and sudden changes, the mental health of college students during the pandemic has attracted widespread attention (12). Sun et al. investigated the mental health of 1912 Chinese college students during the pandemic. They found that 46.55 and 34.73% of college students had symptoms of depression and anxiety, respectively (13). Lockdown and quarantine have led to a decrease in personal physical activity and an increase in sedentary, game-obsessed, and college students' excessive use of electronic devices. Huang et al. found in a survey of 10,357 medical college students that the rate of smartphone addiction was as high as 59.42%, accompanied by sedentary, reduced PA, and poor mental health (10). A study from Spain found that physical activities among college students during the lockdown period of COVID-19 decreased by 29.5 and 18.3% for moderate-intensity and high-intensity PA, respectively. However, the sedentary time increased by 52.7% compared to the period before the pandemic (14). It leads to cognitive impairment, depression negative emotions and other mental health problems. Therefore, it becomes very relevant about how to effectively solve college students' mental health problems during the pandemic.

With the rise of evidence-based research on the relationship between physical activities and mental health, PA showed the association with the rise of general wellbeing and enhanced perception of quality of life and mood, and could significantly alleviate anxiety and depressive symptoms (15, 16). The evidence of a significant and positive association between PA and mental health was also successively and strongly confirmed in COVID-19 pandemic (17–19). A review of previous studies implied that many studies have been conducted to investigate the PA and mental health aspects of general college students during the pandemic (13, 20–22). however, few studies have been conducted on the relationship between PA and mental health of students at sports universities. Students from sports universities belong to a special group, these students usually have better physical quality and habit of participating in sports, and their PA level is higher than that of students from ordinary universities. Some of them take courses that get involved in theoretical and practical aspects of different sports, and they have mastered certain methods of physical exercise and the ability to design training contents (23). Beyond that, some studies found that there is a significant difference in the social adaptability psychological quality between sports college students and non-sports college students, in which sports college students could cope with various stimulis and influences exerted on them by others or the external environment and have greater acceptance and carrying capacity stronger stress coping power (24), Hence, whether such populations were able to engage in regular physical activities during the COVID-19 confinement and whether they had the same mental health problems faced by the average college student should be further explored.

During this outbreak, for positive response to the epidemic prevention policy, Shanghai University of Sport implemented the most strict control measures. In addition to the epidemic control and security personnel, students could not leave the dorms without a special reason. The university was responsible for providing daily meals and deliver to every dorm, so as to reduce the personnel flow and contact, which could minimize the risk of transmission. The strict lockdown limited students' activities to their rooms. They could not process normal travel and exercise, which led to a sharp reduction in physical activity and the possibility of psychological problems that were often seen in mental health studies during the pandemic, such as depression, anxiety, excessive use of electronic products and fear of COVID-19. Therefore, whether the physical education students can process regular physical activities in a limited space and whether they have to face the same mental health problems as the ordinary university students need to be further explored. As a result, the purpose of this study was to conduct a questionnaire survey on undergraduate students at the Shanghai University of Sport during the lockdown period of the pandemic in 2022, in hope of better understanding the current situation of PA and mental health of the students and the association between different physical activity levels and mental health. It will provide basic data reference for sports colleges and universities to carry out health education, PA advice and psychological guidance for college students during the special epidemic period and the post-epidemic period.

## Methods

### Respondent recruitment

In this study, a cross-sectional questionnaire survey of current undergraduate students with confinement was conducted at the Shanghai University of Sport from May 5 to 15, 2022, 4 weeks after strict and comprehensive pandemic control strategies for the whole area. The Shanghai University of Sport is the earliest sports university created after the founding of China, with a variety of disciplines and majors within it, and its Kinesiology program is one of the best sports majors in the country, with many students from athletes having high PA levels and exercise habits. With the consent of the university, this study randomly selected 400 students from six different majors from the 2019 to 2021 grades of Shanghai University of Sport. The selection criteria included: (a) undergraduate students; (b) Not volunteering or working for the university during the pandemic. Compared to graduate students, undergraduate students reported higher rates of emotions and behaviors related to poor mental health (25, 26). While volunteers or staff were required to complete tasks assigned by the university every day, such as meals delivery and nucleic acid testing, etc., which were not suitable for this study.

To guarantee the validity of questionnaires and ensure that respondents were able to understand the content of the questionnaires (detailed instructions are included in the measurement), the study was conducted through the students' teacher in charge who specifically administered and interpreted the questionnaires with an initial sample size of 400 students. After excluding respondents with incomplete and missing data, the information from 376 college students was finally valid.

### Procedure

Considering the safety issues during the pandemic, all respondents completed the questionnaires *via* the WeChat “Questionnaire Star” survey platform with descriptions of the survey instructions. The questionnaires included physical activity scales, depressive symptoms, anxiety symptoms, smartphone addiction, and the fear of COVID-19 scale, as well as some sociodemographic information. The respondents' guardians have been informed about the survey through the respondents' counselors prior to data collection. Subsequently, informed consent forms were sent to the respondents and their parents. After obtaining their consent, the survey was started. The purpose of this study was explained to all respondents and it was emphasized that all data collected would be analyzed in an aggregated manner and that personal information would be kept strictly confidential. This study was based on the Declaration of Helsinki and it was approved by the Ethics Review Committee of Shanghai University of Sport.

### Measurements

#### Exposure

##### Physical activity

Daily PA time and sitting time were self-reported using the International Physical Activity Questionnaire Short Form (IPAQ-SF), which was developed as a surveillance instrument to measure multiple domains of PA (27, 28). The IPAQ-SF is considered a reliable instrument to assay the total amount of PA obtained in the 15–65 years people (28). The IPAQ-SF required respondents to report the frequency and duration of each PA (vigorous-intensity activities, moderate-intensity activities, and walking), as well as the duration of daily sitting during the last 7 days. The Chinese version of the IPAQ-SF has been confirmed adequately reliable and valid in previous studies (27).

#### Outcomes

##### Anxiety symptoms

Anxiety symptoms were measured using the Chinese version of the Generalized Anxiety Disorder scale (GAD-7) (29). Each item has four response options with scores ranging from 0 to 3 (0 = Not at all to 3 = Nearly every day). Each respondent could obtain a total score that ranged from 0 to 21, with a higher score indicating more severe anxiety symptoms. Total scores of 5, 10, 15, and 20 were identified as mild, moderate, moderately severe, and severe anxiety, respectively. Thus, with the cut-off point set at 5, respondents were categorized into two groups: no anxiety symptoms (GAD-7 score < 5) and anxiety symptoms (GAD-7 score ≥5). The Chinese version of GAD-7 has been widely used and well-validated in multiple studies (29), In this study, the Cronbach alpha coefficient of the GAD-7 scale = 0.895 and the correlation coefficients of the GAD-7 and PHQ-9 were 0.751 and 0.934, respectively, with acceptable reliability and convergent validity (30).

##### Depressive symptoms

The Chinese version of the 9-item Patient Health Questionnaire (PHQ-9) was applied to measure the severity of depressive symptoms (31). A total score ranged from 0 to 27 (higher points indicating more severe depressive symptoms), with each item that could earn 0–3 points (0 = Not at all to 3 = Nearly every day). Total scores of 5, 10, 15, and 20 were identified as mild, moderate, moderately severe, and severe depressive symptoms, respectively (31). Thus, a total score of 5 was set as a cut-off point to categorize respondents into two groups: no depressive symptoms (PHQ-9 score < 5) and depressive symptoms (PHQ-9 score ≥ 5). The Chinese version of PHQ-9 has been widely used and well-validated in Chinese adolescents (32), which would be further supported by this study. The Chinese version of the PHQ-9 has been extensively used and validated among Chinese adolescents. The overall reliability coefficients Cronbach s α and McDonald's ω of this scale in this study were 0.824 and 0.86, respectively, and the correlation coefficient between PHQ-9 and GAD-7 was 0.751, implying that its reliability and convergent validity were good (33).

##### COVID-19-related fear

COVID-fear was measured *via* the Chinese version of the Fear of COVID-19 Scale (FCV-19S) (34, 35). It consisted of seven items, with each item that can be responded to on a five-point Likert scale (strongly disagree=1 to strongly agree = 5), with higher scores indicating a greater COVID-fear level. The scale was widely used in several countries, and a systematic review with the evidence from 16 papers including 21 countries and 16 language versions of the FCV-19S found that the different language versions of the FCV-19S were a powerful and valid tool for assessing fear of COVID-19 and did not differ significantly by age and gender (36). Beyond that, the psychometric properties of the FCV-19S have been confirmed in the Chinese population, and some scholars examined the applicability of the FCV-19S in 2,445 Chinese students, and the findings manifested good reliability (reliability and index) where item separation reliability = 1.00, item separation index = 18.44, person separation reliability = 0.88, and person separation index = 2.77. The validity of the FCV-19S was also favorable. The validity of the FCV-19S was also ideal, The mean significant correlation values for FCV-19SC and depression, anxiety, and stress were |r| = 0.467, 0.482, and 0.469, respectively (37). Thus, the FCV-19S-C was shown to be a valid measure of Chinese students' fear of COVID-19. The internal consistency of this study was good, with a Cronbach s alpha coefficient of 0.86, and its construction and criterion validity remained the acceptable limits (38).

##### Smartphone addiction

The Smartphone Addiction Scale short version (SAS-SV) was adopted to measure the extent of smartphone addiction (39, 40). The scale contains 10 items related to smartphone addiction, with a score of 1 to 6 on a scale of “strongly disagree” to “strongly agree” (6 levels). The higher the total score, the higher the degree of smartphone addiction, using ≥33 (females) and ≥31 (males) as their classification criteria for smartphone addiction. A 2019 study showed that the SAS-SV scale was a valid scale for assessing excessive smartphone use among children and adolescents in Hong Kong, and its results indicated that the scale had good convergent validity and the CFA confirmed that the model had acceptable goodness of fit (comparative fit index = 0.96, Tucker Lewis index = 0.95, root mean square error of approximation = 0.06) (41). The scale had good reliability in this study (α = 0.81; ω = 0.78) (42).

### Statistical analyses

Data analysis was performed *via* SPSS 26.0 software. The basic conditions of college students who attended the questionnaire survey were depicted. Then, the descriptive data were expressed as the mean and standard deviation (SD) of continuous variables, and logistic regression equations were used to analyze the association and strength between physical activity and mental health of sports university students with different degrees of physical activity as independent variables, depression, anxiety, fear of COVID-19, and smartphone addiction as dependent variables, respectively, controlling for confounding factors gender, grade, major, family residence, whether they were only children, parents' educational level, and family economic status, and so on, so as to delve into the association and strength between physical activities and mental health of students at sports universities. The test level α = 0.05.

## Results

In this study, a total of 376 questionnaires from students that were around 20 years old (20 ± 1.3) in Shanghai University of Sport were valid, including 144 (38.3%) male students and 232 (61.7%) female students. According to the result, the average scores of long sedentary time (M = 497.4, SD = 524.9), depression (M = 6.7, SD = 5.1), smartphone addiction (M = 34.1, SD = 9.2) were above the normal range. The mean value of anxiety (M = 4.5, SD = 4.4) was kept in the normal range. The average scores in fear of COVID-19 was 2.2 ± 0.8. The daily mean time to perform low to moderate intensity PA was 5.5 (SD = 20.1) min, 12.7 (SD = 23.4) min, and 12 (SD = 21.9) min, respectively, as detailed in Table 1.

**Table 1:** Participants' characteristics.

**Variables**	**Mean or *n* (%)**	**SD**
Sex		
Male	144 (38.3)	
Female	232 (61.7)	
Age (year)	20	1.3
Height (cm)	170.2	12.3
Weight (kg)	64.7	17.3
SED	497.4	524.9
LPA	5.5	20.1
MPA	12.7	23.4
VPA	12.0	21.9
Covid-19 fear	2.2	0.8
Smartphone addiction	34.1	9.2
Depression symptoms	6.7	5.1
Anxiety symptoms	4.5	4.4

LPA, low physical activity; MPA, moderate physical activity; VPA, vigorous physical activity; SED, sedentary behavior.

Binary logistic regression analysis showed that moderate-intensity physical activities was negatively associated with depression (OR = 0.95, 95% CI = 0.93–0.98), anxiety (OR = 0.97, 95% CI = 0.95–0.99), COVID-19 (OR = 0.99, 95% CI = 0.98–0.99) fear and smartphone addiction (OR = 0.94, 95% CI = 0.9–0.98) were negatively associated (all *P*-values < 0.05), and sedentary behavior was positively associated with smartphone addiction (OR = 1.01, *P* < 0.01, 95% CI = 1.001–1.004), after adjusting the model to control for statistically significant characteristic variables such as grade, whether the child was an only child, parental literacy, and family economic status. The results did not change significantly, as detailed in Table 2.

**Table 2:** Associations between LPA, MPA, VPA, and SED with fear of COVID-19, smartphone addition, depressive symptoms and anxiety symptoms.

**Variables**	**Fear of COVID-19**					**Smartphone addiction**					**Depressive symptoms**					**Anxiety symptoms**				
	**β**	***P***	**OR**	**95%CI**		**β**	***P***	**OR**	**95%CI**		**β**	***P***	**OR**	**95%CI**		**β**	***P***	**OR**	**95%CI**	
LPA	0.002	0.414	1.002	0.998	1.006	0.025	0.303	1.025	0.978	1.075	0.003	0.822	0.997	0.972	1.023	0.001	0.9	1.001	0.979	1.025
MPA	−0.005	0.009**	0.995	0.991	0.999	−0.064	0.006**	0.938	0.895	0.982	−0.049	0.000***	0.952	0.929	0.976	−0.03	0.009**	0.971	0.949	0.993
VPA	0.003	0.268	1.003	0.998	1.007	−0.024	0.36	0.976	0.927	1.028	0.011	0.419	1.012	0.984	1.04	0.014	0.264	1.014	0.989	1.04
SED	0.000	0.448	1	1	1	0.002	0.01*	1.002	1.001	1.004	0.001	0.147	1.001	1	1.002	0.001	0.055	1.001	1	1.002

*P < 0.05, **P < 0.01, ***P < 0.001; LPA, low physical activity; MPA, moderate physical activity; VPA, vigorous physical activity; SED, sedentary behavior; CI, confidence interval; OR, odds ratio.

## Discussion

Upholding data from a sample of college students at the Shanghai University of Sport during the confinement period of Shanghai from May 5 to May 15, 2022, the purpose of this study was to determine the relationship between PA and depressive symptoms, anxiety symptoms, COVID-19 fear, and smartphone addiction. It was found that MPA was negatively associated with depressive symptoms, anxiety symptoms, smartphone addiction, COVID-19 fear, and a positive association between sedentary and smartphone addiction. Possible explanations for these results were discussed below.

Shanghai has implemented a series of strict and comprehensive pandemic control strategies to reduce the transmission and infection rate of COVID-19 in 2022. With the purpose of making sure the gathering and contact of people, the university arranged three meals per day by building, time and area during the control period, with uniform delivery to the door of the dormitory, and all school students could not leave the dormitory under the control of the strict policy. The adoption of such a closed management policy may lead to higher levels of depression, anxiety, and stress and lower levels of subjective wellbeing among school students (43). In this context, this study found that students' MPA was negatively associated with depressive symptoms, this result could be supported by previous studies conducted by Herbert which validated the effectiveness of exercise in preventing and reducing depressive symptoms at university students populations, that exercise interventions comprising aerobic exercises of low- to moderate intensity may work best to improve mental health among university students after a few weeks of intervention (44). and the role of moderate-to-vigorous physical activity (MVPA) in alleviating depressive symptoms has also been widely recognized (45–47).

A study showed that during the pandemic, people who regularly engaged in MVPA were 12–32% less likely to experience symptoms of depression. A single 20–60 min session of physical activities 1–5 times per week had the significant positive effect on depression (48) while high-frequency exercise (3–5 times per week) interventions were more effective in alleviating depression symptoms than low frequency (1 time per week) (49). Compared with previous findings, this study realized no significant difference between the effects of low- and high-intensity physical activity on depressive symptoms, and moderate-intensity physical activities were effective in alleviating depression. Some scholars have used restricted cubic spline model analysis to identify an approximate “U” shaped dose-effect relationship between weekly MVPA time, total physical activities and the risk of developing depressive symptoms. With the increase of weekly MVPA time and total physical actibity, the risk of developing depressive symptoms showed a tendency to decrease then increase (50). It could be seen that exercise intensity and total physical activities should be maintained at an appropriate intensity and level for alleviating and preventing the occurrence of depressive symptoms (51); another important reason may be that it was difficult to perform strenuous physical activities during the closure control period due to the policy of preventing from leaving home and the lack of corresponding exercise equipment for college students in physical education (17), and moderate-intensity exercises, such as jumping gymnastics, self-weight strength training and other moderate-intensity physical activities are commonly chosen by students and have produced good alleviating effects on depression.

In this study, MPA was also negatively associated with anxiety symptoms, a result was consistent with a large body of research and showed that physical activities had a positive effect on improving mood and wellbeing, reducing anxiety and promoting mental health (52). From the neurobiological mechanism hypothesis, PA enhanced mental health and alleviated anxiety symptoms by changing the structural and functional organization of the brain (53) while the behavioral mechanism hypothesis suggested that physical activities improved self-regulation and coping skills, thus helping people to stay mentally active effectively (54). Physical activities of appropriate intensity and frequency could release psychological tension and increase mental stability (55). In 2020, the World Health Organization gave more specific recommendations that for adults aged 18–64 years, at least 150 min of moderate-intensity aerobic exercise or at least 75 min of high-intensity aerobic exercise per week may reduce the risk of anxiety (56). Promoting mental health by maintaining and improving PA levels have already became a critical and highly effective measure with effects comparable to those of medication and psychotherapy, and it is widely recognized (56–58).

Prolonged lockdown and removal from the constraints of the normal group environment may be accompanied by changes in various behavioral habits of college students, including the frequency of smartphone use. Smartphone addiction is a new type of behavioral addiction in which individuals use smartphones excessively and have no control over the behavior, resulting in impaired social functioning and bringing about psychological and behavioral problems (59). In this study, the average score of smartphone addiction for the sample as a whole was 34 (over 33 for female students and over 31 for male students as having symptoms of smartphone addiction), implying that most students are in a state of smartphone addiction during the epidemic control period, just like students who came from general universities, and that smartphone addiction might affect physical activities during the day (60, 61). This study found that students engaging in moderate-intensity PA were negatively associated with smartphone addiction, and sedentary behavior were positively linked with smartphone addiction. A study last year has the similar results, in which smartphone addicted college students resulted in the increased sedentary time and decreased physical activity time compared to the pre-COVID-19 outbreak. It meant that long sedentary time and low moderate physical activity time were positively associated with smartphone addiction among college students (10). Also, a 2020 US study also showed that during the early restriction period of COVID-19, a reduction in PA and increased time spent using smartphone screens led to poorer mental health outcomes (62), the longer the sedentary time was, the poorer mental health and wellbeing would be (9). This study validated previous studies in some respects that performing moderate-intensity PA can reduce the level of smartphone addiction, and that sedentary behavior is a risk factor for smartphone addiction. Therefore, it can be suggested that reducing sedentary behaviors and maintaining moderate to high PA levels during self-quarantine of the COVID-19 pandemic should be an important measure to avoid smartphone addiction and promote physical and mental health among college students.

Furthermore, this study found a negative correlation between MPA and fear on the COVID-19, which is also consistent with previous studies that found a negative correlation between fear scores and physical activity levels on the COVID-19 (higher fear was associated with lower physical activity levels), with each increase in scale score decreasing the odds of engaging in strenuous physical activity 3% (63). Engaging in moderate-intensity physical activities contributes to and alleviates fear of COVID-19 and can be explained by theories of stress and maladaptive coping as well as health behavior theories because COVID-19 global spread exceeds an individual's psychosocial resources (e.g., social support; self-regulatory skills) and exacerbates an individual's fear of COVID-19 and thus may be reduced by using maladaptive coping strategies to respond, thereby reducing time spent participating in physical activities.

In interpreting the results of the above study, some limitations of this study were noted. First of all, this study made use of a cross-sectional study design, which only allowed for correlational analysis and did not allow for causal inference, and it was more difficult to collect questionnaires and had a smaller sample size during the school closure period of the pandemic. Future studies should be able to consider larger sample sizes and use longitudinal designs and randomized controlled trials to help establish causal relationships between physical activities, depression, anxiety symptoms, smartphone addiction, and fear of COVID-19. Another limitation of this study was the self-reported nature of the data, which was heavily influenced by potential recall bias and current subjective mood. For example, people who have experienced poor mental health problems may be more inclined to participate in research because they find the topic more relevant. This may lead to an overestimation of depressive and anxiety symptoms. Future studies should use more objective measures. Furthermore, the mechanisms associated with why only moderate-intensity PA can be a significant influence on depression, anxiety symptoms, smartphone addiction, and fear of COVID-19 factors in this study remained an open question for future research.

Despite these limitations, our findings still have clinical and policy implications. First of all, university psychology counselors could further evaluate the psychological problems of the students based on the result of the investigation, in order to implement effective intervention, which will help students to alleviate mental health problems in the lockdown. In addition, moderate intensity exercise seems to be a better choice in the relatively small space. The students could choose to do some self-weight strength training, jumping gymnastics, rope skipping and other moderate-intensity physical activities (64). It is recommended to have exercises for 20 min a day, adjust life schedule and reduce sedentary behaviors, so as to alleviate mental health problems caused by the epidemic lockdown.

## Conclusion

There was a significant association between moderate-intensity physical activities and depression, anxiety, fear of COVID-19, and smartphone addiction, and a significant association between sedentary behavior and smartphone addiction among sports college students during the lockdown. The findings again demonstrated that physical activities and reduction of sedentary behavior could contribute to the mental health of college students, but further longitudinal studies should be needed to elucidate the causal relationship between moderate-intensity physical activities and mental health among sports college students. This study suggested that during the pandemic, current college students in closure management should be encouraged to overcome difficulties such as space limitations, actively engage in moderate-intensity physical activity, and reduce sedentary behaviors, which are beneficial for alleviating mental health problems, such as depression, anxiety symptoms, fear of COVID-19, and smartphone addiction.

## Data availability statement

The original contributions presented in the study are included in the article/supplementary material, further inquiries can be directed to the corresponding author/s.

## Ethics statement

The studies involving human participants were reviewed and approved by Ethics Review Committee of Shanghai University of Sport. The patients/participants provided their written informed consent to participate in this study.

## Author contributions

YW: conceptualization, methodology, investigation, data curation, and writing—original draft. YL: validation, supervision, formal analysis, writing—reviewing and editing, and funding acquisition. Both authors contributed to the article and approved the submitted version.

## Conflict of interest

The authors declare that the research was conducted in the absence of any commercial or financial relationships that could be construed as a potential conflict of interest.

## Publisher's note

All claims expressed in this article are solely those of the authors and do not necessarily represent those of their affiliated organizations, or those of the publisher, the editors and the reviewers. Any product that may be evaluated in this article, or claim that may be made by its manufacturer, is not guaranteed or endorsed by the publisher.
